# Discovery of Highly Active Recombinant PNGase H^+^ Variants Through the Rational Exploration of Unstudied Acidobacterial Genomes

**DOI:** 10.3389/fbioe.2020.00741

**Published:** 2020-07-03

**Authors:** Rui-Rui Guo, Gerard Comamala, Huan-Huan Yang, Marius Gramlich, Ya-Min Du, Ting Wang, Anne Zeck, Kasper Dyrberg Rand, Li Liu, Josef Voglmeir

**Affiliations:** ^1^Glycomics and Glycan Bioengineering Research Center (GGBRC), College of Food Science and Technology, Nanjing Agricultural University, Nanjing, China; ^2^Protein Analysis Group, Department of Pharmacy, University of Copenhagen, Copenhagen, Denmark; ^3^Natural and Medical Sciences Institute (NMI), University of Tubingen, Reutlingen, Germany

**Keywords:** acidic PNGase, *N*-glycans, glycoprotein deglycosylation, *Dyella japonica*, analytical glycoscience

## Abstract

Peptide-*N*^4^-(*N*-acetyl-β-glucosaminyl) asparagine amidases (PNGases, *N*-glycanases, EC 3.5.1.52) are indispensable tools in releasing *N*-glycans from glycoproteins. So far, only a limited number of PNGase candidates are available for the structural analysis of glycoproteins and their glycan moieties. Herein, a panel of 13 novel PNGase H^+^ candidates (the suffix H^+^ refers to the acidic pH optimum of these acidobacterial PNGases) was tested in their recombinant form for their deglycosylation performance. One candidate (originating from the bacterial species *Dyella japonica*) showed superior properties both in solution-phase and immobilized on amino-, epoxy- and nitrilotriacetate resins when compared to currently acidic available PNGases. The high expression yield compared to a previously described PNGase H^+^, broad substrate specificity, and good storage stability of this novel *N*-glycanase makes it a valuable tool for the analysis of protein glycosylation.

## Introduction

The study of protein N-glycosylation is an active area of biotechnological research, given that all antibodies, and many biotherapeutics as well as extracellular and cell surface proteins bear these post-translational modifications (Overington et al., 2006; Saldova et al., 2017). The detailed analysis of therapeutic glycoproteins is of great importance, as the quality and effectiveness of these drugs strongly depend on the consistency of their glycan moieties (Berkowitz et al., 2012; Duivelshof et al., 2019). Peptide-*N*^4^-(*N*-acetyl-β-glucosaminyl) asparagine amidases (PNGases) enzymatically release asparagine-linked glycan moieties from glycoproteins and have been described as valuable tools in the analysis of glycotherapeutics (Wang and Voglmeir, 2014). A subset of these PNGases used in bioanalytical applications (namely PNGase A and PNGase At) originate from plants and show a peculiar promiscuity toward a broad range of eukaryotic glycoproteins, including those from plants and insects (Takahashi, 1977; Altmann et al., 1998; Yan et al., 2018). Furthermore, the low pH optimum of these enzymes is of particular interest for analytical methods which require acidic conditions, such as rapid N-glycans derivatization methods (Du et al., 2015) or hydrogen-deuterium exchange mass spectrometry (HDX-MS) of glycoproteins (Jensen et al., 2016).

One limitation of PNGase A (and based on the close homology, also PNGase At) is that this enzyme itself is a glycoprotein extracted from almonds (Takahashi, 1977), thus it can undergo auto-deglycosylation, thereby contaminate analyzed samples with endogenous PNGase A glycan structures (Altmann et al., 1998). Moreover, there are currently no reports describing the successful recombinant expression of PNGase A in prokaryotic systems, and therefore limiting the use of engineered fusion tags for the facilitated purification and immobilization of this enzyme. Interestingly, our research group described recently a PNGase A homologue from Acidobacteria instead of plants (Wang et al., 2014). This phylum of soil bacteria was virtually unknown until 2009 (Ward et al., 2009). The discovered PNGase originated from *Terriglobus roseus* and was recombinantly expressed in *E. coli*, and due to its surprisingly low pH optimum of pH 2.5 designated as “PNGase H^+^.” Although the enzymatic activity of PNGase H^+^ from *Terriglobus roseus* could be successfully used for conventional N-glycan analysis protocols (Wang et al., 2017; Du et al., 2018), its use in a wider range of analytical workflows was hampered by difficulties in optimizing expression and purification of the enzyme at larger scales. With the rapid progress and the availability of whole-genome sequences in recent years, >70 acidobacterial PNGase H^+^ homologs can be currently found using BLAST (Altschul et al., 1990) in March 2020. This pool of candidate genes, therefore, allows the evaluation of alternative PNGase candidate genes for their biotechnological potential. In this *Brief Research Report*, we studied the activity and stability of 12 unstudied acidobacterial PNGase H^+^ candidates and we compared them to the previously described PNGase from *Terriglobus roseus*, both in solution and immobilized on various solid supports.

## Materials and Methods

### Materials

Epoxy- and amino activated methacrylate beads were kindly donated by Purolite (Zhejiang, China). Ni-NTA (nickel-ion nitrilotriacetic acid) superflow resin was obtained from Qiagen (Shanghai, China). WST-1 (sodium 2-(4-iodophenyl)-3-(4-nitrophenyl)-5-(2,4-disulfophenyl)-2H-tetrazolate) was purchased from Sangon Biotech Company (Shanghai, China). Horseradish peroxidase (HRP) was obtained from Duly Biotech Ltd. (Nanjing, China). Human IgG was purchased from ProteinTech (Nanjing, China). Lactoferrin was obtained from Wako Pure Chemical Industries (Nanjing, China). PNGase F was provided by Qlyco Ltd. (Nanjing, China). All other standard chemicals and buffer reagents were of the highest grade available.

### Construction of the Recombinant Plasmids

The genes encoding the putative PNGase homologs Ac (from *Acidobacterium capsulatum*, UniProt ID C1F2K4) and Ka (from *Kutzneria albida*, UniProt ID W5W1E9) were amplified from bacterial DNA and cloned into a pET30a expression vector as described for the PNGase Tr (from *Terriglobus roseus*, UniProt ID I3ZL27) previously (Wang et al., 2014). The other 10 candidate genes were synthesized in *Escherichia coli* K12 codon preference and ligated on the pET30a vector by Genscript Ltd. (Nanjing, China) and designated as PNGases Le (from *Lysobacter enzymogenes*, UniProt ID A0A0S2DLB6), Sp (from *Streptomyces populi*, UniProt ID A0A2I0SFR1), Sa (from *Streptomyces albulus*, UniProt ID A0A059W230), Ss (from *Solimicrobium silvestre*, UniProt ID A0A2S9GXY8), Sb (*Silvibacterium bohemicum*), Ab (from *Acidobacteria bacterium*, GenBank ID PYX41174), Ea (from *Edaphobacter aggregans*, GenBank ID WP_035357016), Xt (*Xanthomonass theicola*, GenBank ID PPT80182), Ft (from *Frateuria terrea*, UniProt ID A0A1H6SY22), and Dj (from *Dyella japonica*, UniProt ID A0A075JY55).

### Expression, Purification, and Immobilization of Recombinant PNGases

The expression and Ni-NTA affinity purification of the 13 PNGase candidate genes were performed as previously described for the *Terriglobus roseus* homolog (Wang et al., 2014). The enzymes’ activities were evaluated using both a gel-based deglycosylation assay (section “Gel-Based Deglycosylation Assay”) and a microplate-based activity test (section “Microplate-Based Activity Assay”). The most active PNGase (Dj) was selected for detailed biochemical characterization and for immobilization studies.

For the immobilization using amino-functionalized methacrylate resins (ECR8305F and ECR8310F) or the epoxy-activated methacrylate resins (ECR8214F and ECR8205F), 500 mg of each resin was equilibrated in immobilization buffer (0.5 M NaCl, 50 mM sodium phosphate buffer, pH 8.0). Then, 4 mL of each purified enzyme solution was mixed with the resins and incubated at 4°C for 18 h with orbital shaking. Then, the mixture was filtered and washed with an immobilization buffer. For the immobilization of PNGase Dj on Ni-NTA resin, 500 mg aliquots were removed after the washing step of the affinity purification. The immobilization efficiency was determined by measuring the PNGase activity of either the purified enzyme solutions (20 μL), the supernatants of the immobilization reactions (20 μL), or the supporting resins (50 mg). In all cases, the immobilization yield (Ψ) was calculated as follows:

$$\Psi \left(\%\right)=\frac{{\text{ABS}}_{\text{initial}}-{\text{ABS}}_{\text{supernatant}}}{{\text{ABS}}_{\text{initial}}}\times 100$$

where ABS_initial_ is the determined absorbance values at 582 nm using the microplate-based activity assay of the purified PNGase solutions offered to the resins, while ABS_supernatant_ is the absorbance values of the remaining PNGase proteins that remain in the supernatant after the immobilization time.

### Gel-Based Deglycosylation Assay

The enzymatic activity of the PNGase candidates was evaluated using a gel-based deglycosylation of horseradish peroxidase (HRP) previously described (Wang et al., 2014). A 20 μL assay mixture consisting of 5 μL of purified PNGase enzyme solution, 1 μL of HRP substrate (10 μg/μL, denatured at 95°C for 10 min), 6 μL of sodium phosphate-citrate buffer (1 M, pH 2.0) and 8 μL of water was incubated at 37°C for 16 h. For the deglycosylation experiments of Lactoferrin or human IgG, 5 μg of the dried glycoproteins were either incubated with 20 μL the PNGase Dj reaction mixture or with 20 μL of a PNGase F reaction mixture (consisting of 0.1 μg of PNGase F and 150 mM sodium phosphate buffer (pH 7.5) in water). Then the assay mixtures were mixed with 5 μL of Laemmli denaturation buffer (fivefold concentrated) and incubated at 95°C for 10 min. These samples were then separated by sodium dodecyl sulfate-polyacrylamide gel electrophoresis (SDS-PAGE) and protein bands visualized by Coomassie Brilliant Blue R-250 staining.

### Microplate-Based Activity Assay

This rapid colorimetric activity test relies on the reduction of the yellowish tetrazolium dye WST-1 into a blue formazan dye by the reducing end of N-glycans, which were enzymatically released from glycoprotein substrates (Wang et al., 2019). Typically, reaction mixtures of 20 μL contained 5 μL of purified PNGase enzyme solution, 5 μL of HRP substrate (20 μg/μL, denatured at 95°C for 10 min), 6 μL of sodium phosphate-citrate buffer (1 M, pH 2.0), and 4 μL of distilled water. For negative controls purified PNGase protein solutions were replaced with water. Samples were incubated at 37°C for 3 h. Then the reaction was quenched by adding 20 μL of 2.5 M trichloroacetic acid, samples centrifuged at 12,000 g for 30 min, and 10 μL of the supernatants transferred into a 384-well microplate. After adding 5 μL of NaOH solution (4 M) and 10 μL of aqueous WST-1 solution (1.7 mM), the mixture was incubated at 50°C for 1 h and subsequently analyzed at a wavelength of 584 nm using a microplate reader (Thermo Multiscan FC, Shanghai, China).

### Temperature Effects and Reusability of PNGase Dj

The following parameters were measured using the microplate-based activity assay described in section “Microplate-Based Activity Assay.” The optimum temperature of PNGase Dj was determined by incubating the reaction mixtures at various temperatures ranging from 25 to 70°C. For testing the storage stability of the enzyme, the purified PNGase solution was stored for up to 60 days at 4°C, and the activity of sample aliquots tested in 7–15 day intervals. To assess the short-time thermal stability of the PNGase, enzyme samples were incubated at temperatures between 22 and 60°C for up to 24 h, and then used for activity tests. The recycling abilities of the immobilized enzyme were studied by reusing the resins in consecutive reactions. Therefore, 100 mg of enzyme carriers were incubated for 30 min at 37°C with 500 μL recycling buffer (consisting of 50 mM sodium phosphate-citrate buffer (pH 2.0), with or without 0.5% (w/v) BSA) and HRP (5 μg/μL) on a rotation mixer. After centrifugation (1 min, 2000 g), 30 μL from the supernatant was used for the microplate-based activity assay. The rest of the supernatant was discarded, and the resin washed twice with 500 μL recycling buffer. Then, the washed resin was reused again for a total of six cycles.

### Analysis of N-Glycans

For the N-glycan release from mouse serum, 50 μL of the serum were incubated with either 150 μL of PNGase F reaction mixture [consisting of 50 μg of PNGase F, 0.15 % (w/v) of SDS, 0.25% (v/v) of 2-mercaptoethanol, 1.25% (w/v) of Triton X-100, and 75 mM of sodium phosphate buffer (pH 7.5) in water], or 150 μL of PNGase Dj reaction mixture [consisting of 50 μg of PNGase Dj and 470 mM of citrate/sodium phosphate buffer (pH 2.0) in water]. The deglycosylation reactions of Lactoferrin or human IgG were performed as described in section “Gel-Based Deglycosylation Assay.” After incubating the reaction mixtures for up to 16 h at 37°C, the samples were centrifuged for 5 min at 12,000 g and the supernatant subject to solid-phase extraction (Supelclean ENVI-Carb, 0.3 mL bed volume, prewashed with 1 mL of water, Supelco). After the samples were washed with 3 mL of water, the enzymatically released N-glycans were eluted from the SPE column using 3 mL of aqueous 40% acetonitrile solution containing 0.1% TFA (v/v/v). Then, the elution fractions were dried by centrifugal evaporation and subject to fluorescence derivatization using 10 μL of a 2-aminobenzamine (2-AB) labeling solution [consisting of 35 mM of 2-AB and 0.1 M of sodium cyanoborohydride in a dimethyl sulfoxide/acetic acid solution (7:3, v/v)]. After 2 h of incubation at 65°C, 5 μL of the sample was mixed with 70 μL of water and 175 μL of acetonitrile, and 40 μL of this sample solution was injected into a HILIC-UPLC system (hydrophilic interaction ultra-performance liquid chromatography, Nexera, Shimadzu) equipped with an Acquity BEH Glycan column (1.7 μm, 130Å, 2.1 × 150 mm, Waters) at an initial flow rate of 0.5 mL/min. The analytes were separated using aqueous ammonium formate (50 mM, pH 4.5) and 100% acetonitrile as mobile phases (solvent A and B, respectively). A linear gradient of 95–78% of B was applied from 0 to 6 min, and B was then decreased to 55.9% over 44.5 min. The analytes were monitored by fluorescence detection (Ex: 330 nm, Em: 420 nm) and were annotated using the GlycoBase N-glycan repository (Campbell et al., 2008). The MALDI-ToF MS analysis of underivatized HRP-derived N-glycans was performed with a crude PNGase Dj assay solution by mixing 1 μL of the sample solution and 1 μL of 6-aza-2-thiothymine (ATT) solution (consisting of 0.3% w/v in 70% v/v aqueous acetonitrile), and using a Bruker Autoflex Speed Mass Spectrometer in positive ion mode.

## Results and Discussion

### Evaluation of Acidobacterial PNGase Activity

**FIGURE 1:**
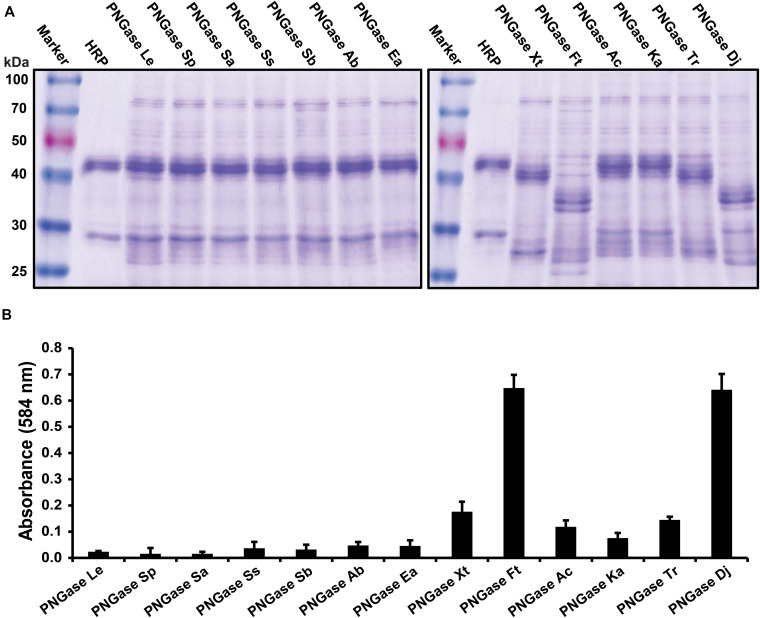
Activity tests of the PNGase candidate genes. **(A)** Gel-based deglycosylation assay to observe the molecular weight shift of the HRP glycoprotein band using SDS-PAGE. Deglycosylation of HRP is seen by a shifting of the major HRP protein band from approximately 45 kDa down to approximately 35 kDa. **(B)** Microplate-based activity test. The data are represented as the mean values and the error bars showing the standard deviation of three independent activity assays.

Twelve putative PNGases candidate genes were selected from data from 32 publicly available acidobacterial genomes by choosing the most distinct homologs of this pool, so that the individual amino acid similarities were below 70% for each PNGase candidate. After expression, the purified enzyme solutions were subjected to the gel-based deglycosylation assay (Figure 1A) and the microplate-based activity test (Figure 1B). Both tests showed that PNGase homologs Xt, Ac, and Ka had a comparable deglycosylation activity to the previously described PNGase Tr, whereas homologs Ft and Dj exhibited increased deglycosylation of HRP relative to all other PNGases. Furthermore, the platereader assay showed that the overall deglycosylation activity of Ft and Dj was approximately fivefold higher compared to PNGase Tr. Other PNGase candidate genes showed only negligible deglycosylaton activities. Given the high activity of the PNGase Dj homology, compared to PNGase Tr, a detailed biochemical characterization and the immobilization trials focused on this enzyme.

### Purification and Immobilization

**FIGURE 2:**
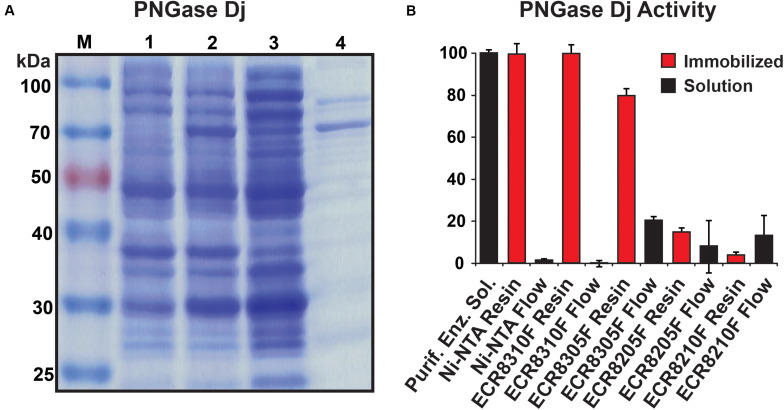
**(A)** SDS-PAGE analysis of recombinant PNGase Dj; M: protein size marker, lane 1: cells before induction, lane 2: cells after induction, lane 3: supernatant from cell lysate, lane 4: purified enzyme. **(B)** PNGase Dj activity in immobilized (red) and soluble form (black). The data are represented as the mean values and the error bars showing the standard deviation of three independent activity assays.

The purified PNGase Dj protein sample migrated as a dominant protein band with a molecular weight of approximately 65 kDa (Figure 2A). The mass of PNGase Dj was further examined using liquid chromatography and ESI mass spectrometry (LC-MS, Supplementary Figure 1), and the identity of the protein sequence was verified by LC-MS/MS analysis of peptides following digestion of PNGase Dj with Trypsin and Pepsin (Supplementary Figures 2, 3). Interestingly the MS data suggest that there is a discrepancy of 2849 Da between the expected theoretical protein mass and the measured protein mass (expected: 64417 Da, observed 61568 Da). This discrepancy can be best explained by the proteolytic cleavage of the first 28 N-terminal residues during expression (theoretical mass 61572 Da, 63 ppm deviation from the measured mass).

PNGase Dj was chosen for immobilization, and different enzyme carrier resins (ECRs) with different immobilization chemistries were evaluated by measuring the immobilization yield (Ψ). The Ψ values for all five tested enzyme carriers were determined to be Ψ_Ni–NTA_ = 99%, Ψ_ECR8310F_ = 100%, Ψ_ECR8305F_ = 80%, Ψ_ECR8205F_ = 92%, and Ψ_ECR8310F_ = 87%. Despite these relatively high immobilization yields, only the Ni-NTA and the amino carrier resins ECR8305F and ECR8310F showed high enzymatic deglycosylation activities in the range between 75 and 95% of the enzyme in-solution activity (Figure 2B). Conversely, and despite the successful enzyme immobilization, the epoxy resins ECR8214F and ECR8205F displayed lower activities of 13 and 4% in comparison to in-solution enzyme activity. We presume that the unspecific nucleophilic reaction of the epoxy groups on the resins with lysine, histidine, cysteine, and tyrosine residues in PNGase Dj may be the reason for the inactivation of the enzyme. By using the glutaraldehyde-activated amino resins ECR8305F and ECR8310F, which specifically target lysine residues for the formation of Schiff bonds, the covalent immobilization of PNGase Dj could be achieved with high yield. Due to the simplicity of the non-covalent immobilization on Ni-NTA resins, the use of this carrier was the most promising for experimental setups where no covalent attachment of the PNGases is required.

### Enzyme Characteristics, Temperature Stability, and Reusability

**FIGURE 3:**
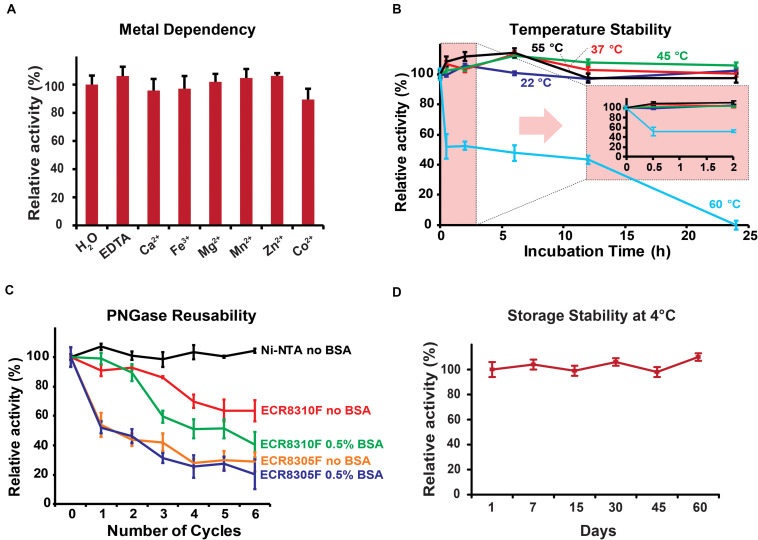
**(A)** Metal ion dependency of PNGase Dj. **(B)** Thermal stability of PNGase Dj at various temperatures. **(C)** Reusability of PNGase Dj using various resins. **(D)** Long-time storage stability of PNGase Dj at 4°C. The data are represented as the mean values and the error bars showing the standard deviation of three independent activity assays.

The biochemical parameters of PNGase Dj were generally comparable to the previously studied Tr homolog: Dj showed the highest activity in the pH range <3.5 with an optimum at pH 2 (Supplementary Figure 4), a temperature optimum of 55°C (Supplementary Figure 5), and its independence of metal ions (Figure 3A). The analysis of the released N-glycans from HRP using HILIC-UPLC (Supplementary Figure 6A) and MALDI-ToF mass spectrometry (Supplementary Figure 6B) showed that PNGase Dj has a comparable substrate specificity with previously described acidic PNGase homologs (Wuhrer et al., 2005; Wang et al., 2014). PNGase Dj was then used to deglycosylate bovine lactoferrin and human IgG, resulting in the complete deglycosylation of these mammalian glycoproteins within 6 h (Supplementary Figure 7). The HILIC-UPLC analysis showed a comparable N-glycan distribution as described in the literature (Reusch et al., 2015; Zhang et al., 2019), confirming that N-glycans bearing α1,6-fucosylation can be also released by this enzyme (Supplementary Figure 8). The effect of the acidic reaction conditions on acid-labile sialic acid moieties was also evaluated by releasing highly sialylated mouse serum glycoproteins using PNGase Dj, and comparing the resulting N-glycans with samples treated with PNGase F at neutral pH (pH 7.5, Supplementary Figure 9). The results showed that even after 16 h of incubation, the loss of sialylation affected mainly tetrasialylated N-glycan species, whereas the sialylation of mono-, di- and tri-sialylated N-glycans was less affected. Given that these experiments were performed at 37°C, we presume that lowering the reaction temperature would allow us to reduce the loss of sialic acids further. The recombinant PNGase was able to maintain full activity for at least 24 h of incubation at temperatures up to 55°C, but almost completely lost activities when incubated for 24 h at 60°C (Figure 3B). The reusability of the PNGase Dj enzyme immobilized on various resins showed that the Ni-NTA carrier could be reused for six cycles without loss of activity, whereas the amino resins ECR8305F and ECR8310F showed activity losses between 10 and 25% per cycle (Figure 3C). Furthermore, no PNGase activities were detected in the acidic reaction buffer, indicating the stable immobilization of PNGase Dj on the Ni-NTA resin (Supplementary Figure 10). No improvements could be achieved when 0.5% BSA (w/v) was added to the reaction mixtures. The storage stability of PNGase Dj was defined as relative activity between the first and successive reactions; No activity losses of the enzyme were observed during the test period of 60 days when stored at 4°C (Figure 3D).

## Conclusion

The research fields of glycomics and glycoproteomics are developing rapidly. Therefore, the discovery of robust and highly active PNGases is key to further improve these technologies. The evaluation of the herein presented PNGase homolog from *Dyella japonica* in solution and on various solid supports provides a solid foundation for future applications of these enzymes. The establishment of robust and user-friendly protocols using immobilized PNGase Dj for various analytical applications including hydrogen-deuterium exchange mass spectrometry (HDX-MS) of highly glycosylated proteins, or the proteomic identification of glycoproteins after deglycosylation is the current endeavor of our research groups.

## Data Availability Statement

The raw data supporting the conclusions of this article will be made available by the authors, without undue reservation, to any qualified researcher.

## Author Contributions

R-RG, GC, H-HY, MG, and Y-MD performed the experiments. R-RG, GC, AZ, KR, LL, and JV analyzed the data. R-RG, GC, KR, LL, and JV wrote the manuscript. LL and JV conceived the study. AZ, KR, LL, and JV coordinated the project. All authors contributed to the article and approved the submitted version.

## Conflict of Interest

The authors declare that the research was conducted in the absence of any commercial or financial relationships that could be construed as a potential conflict of interest.
