# Supplementary material for: Discovery of Highly Active Recombinant PNGase H+ Variants Through the Rational Exploration of Unstudied Acidobacterial Genomes
Source: Front Bioeng Biotechnol. 2020 Jul 3;8:741. doi: 10.3389/fbioe.2020.00741 (PMC7348039; doi:10.3389/fbioe.2020.00741)
Supplement: Supplementary file 1 [file Image_1.pdf]

Supplementary Material

Discovery of highly active recombinant PNGase H$^+$ variants through the rational exploration of unstudied acidobacterial genomes

Rui-Rui Guo, Gerard Comamala, Huan-Huan Yang, Marius Gramlich, Ya-Min Du, Ting Wang, Anne Zeck, Kasper Dyrberg Rand, Li Liu, and Josef Voglmeir

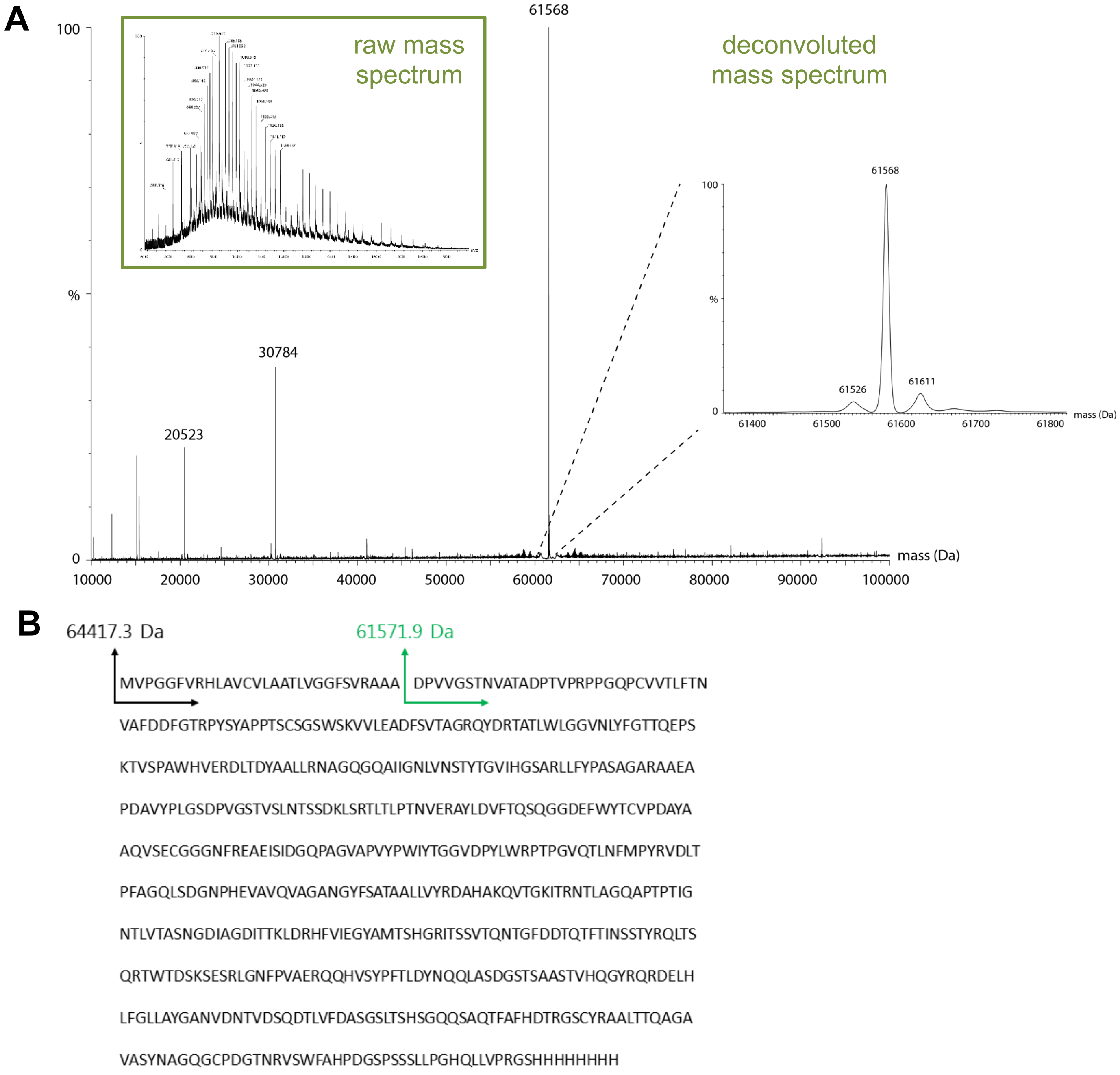

Supplementary Figure 1. (A) Deconvoluted and raw ESI mass spectrum of PNGase Dj. (B) Protein sequence of full length PNGase Dj (black arrows) and the predicted N-terminally truncated PNGase sequence (green arrows).

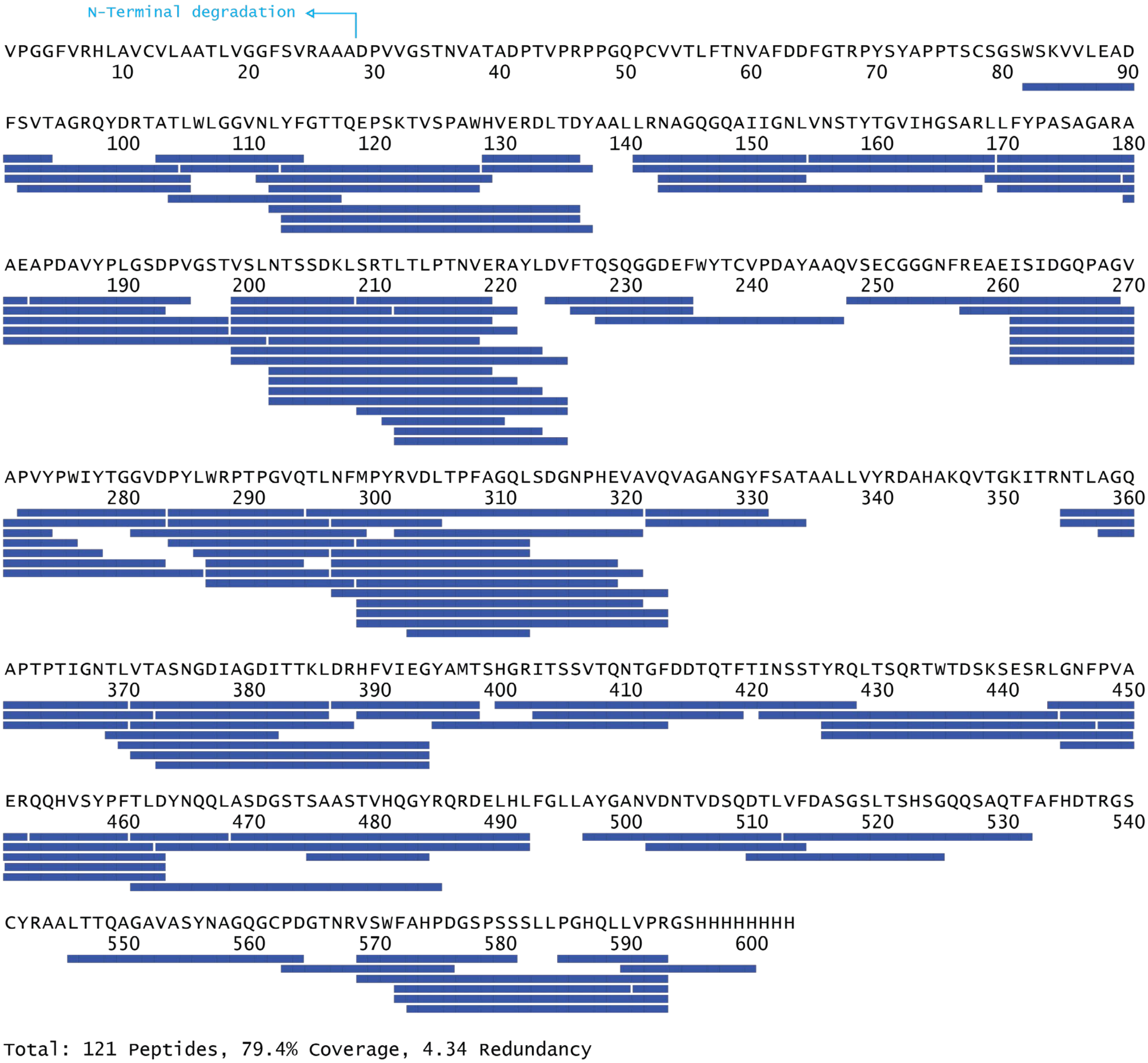

Supplementary Figure 2. Map of identified peptides of PNGase Dj following pepsin digestion and LC-MS/MS analysis.

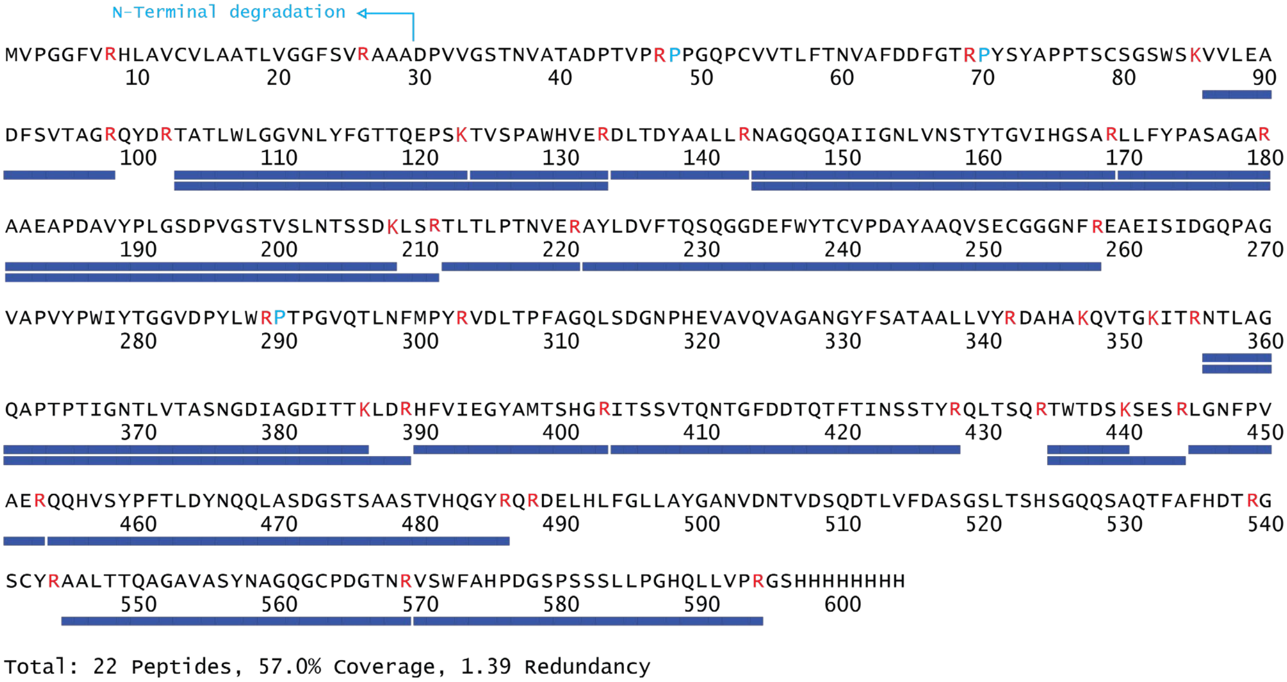

Supplementary Figure 3. Map of identified peptides of PNGase Dj following trypsin digestion and LC-MS/MS analysis.

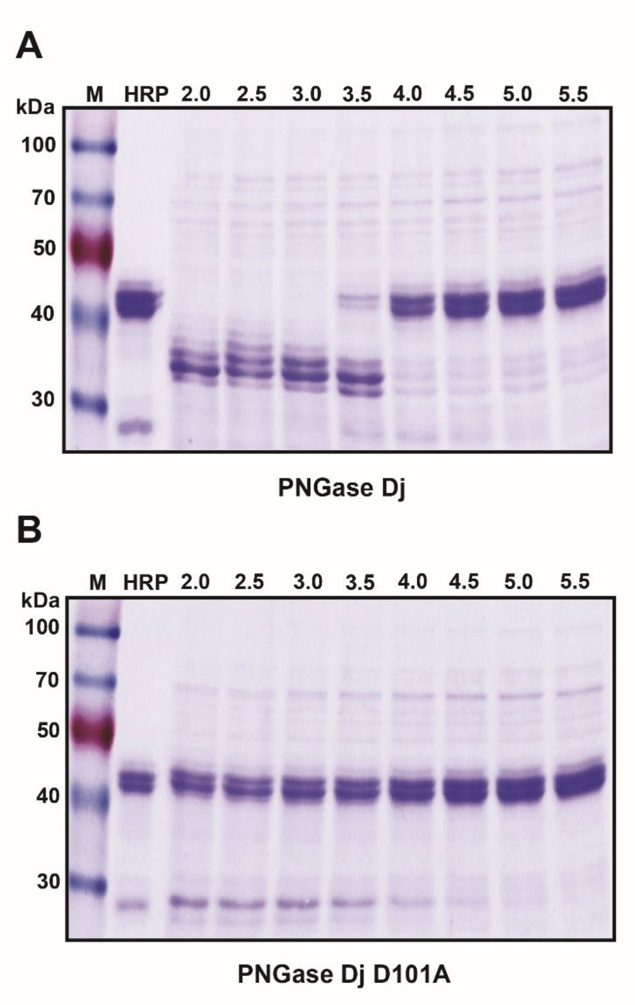

Supplementary Figure 4. Determination of the pH optima of PNGase Dj **(A)** using a gel-based deglycosylation assay to observe the molecular weight shift of the HRP glycoprotein band upon treatment with the PNGase enzymes. Control experiment using inactive PNGase mutant variant Dj D101A **(B)**.

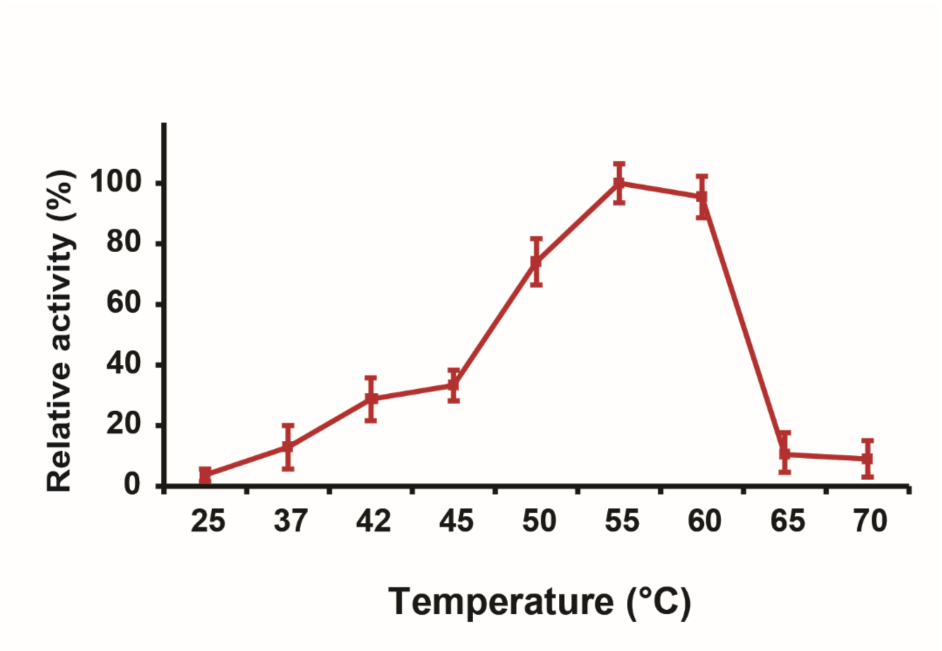

Supplementary Figure 5. Temperature optimum of PNGase Dj. The data are represented as the mean values and the error bars showing the standard deviation of three independent activity assays.

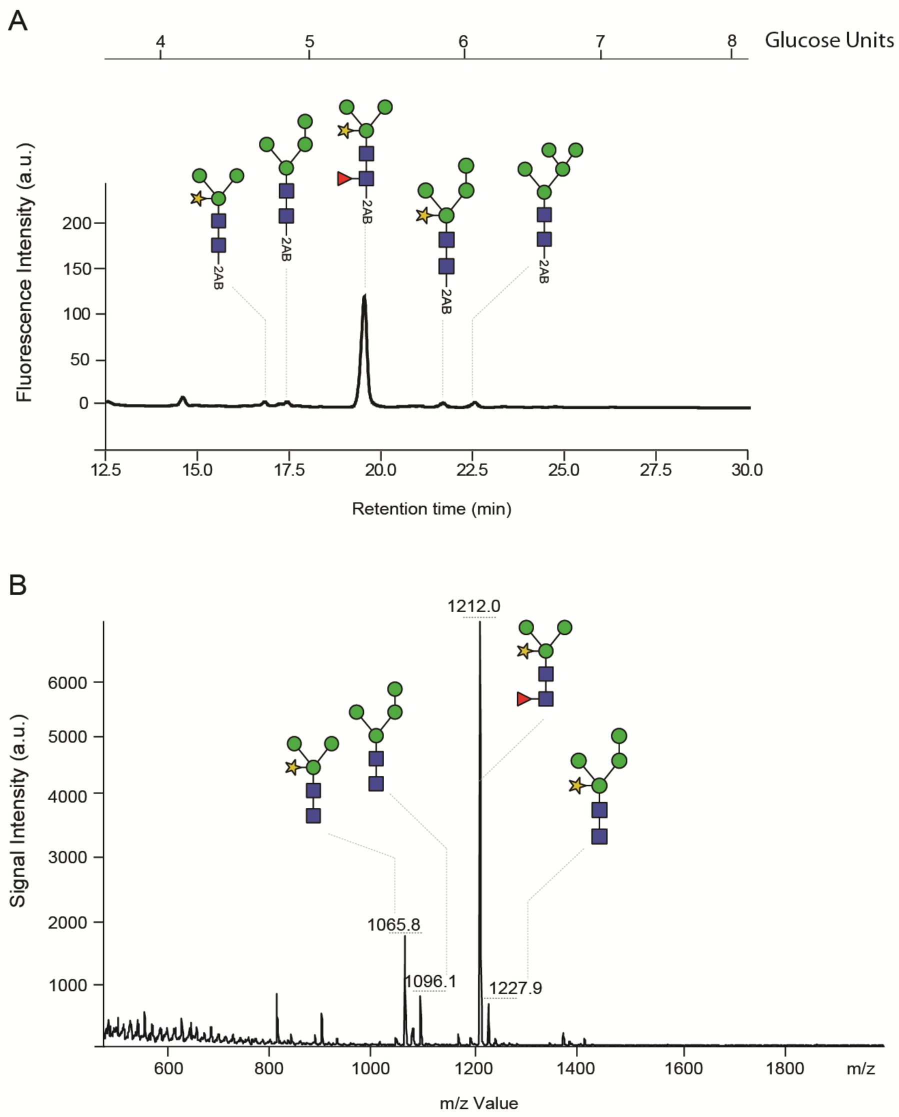

Supplementary Figure 6. Analysis of N-glycans derived from Horseradish Peroxidase (HRP) after PNGase Dj treatment using HILIC-UPLC **(A)** and MALDI-ToF Mass Spectrometry **(B)**.

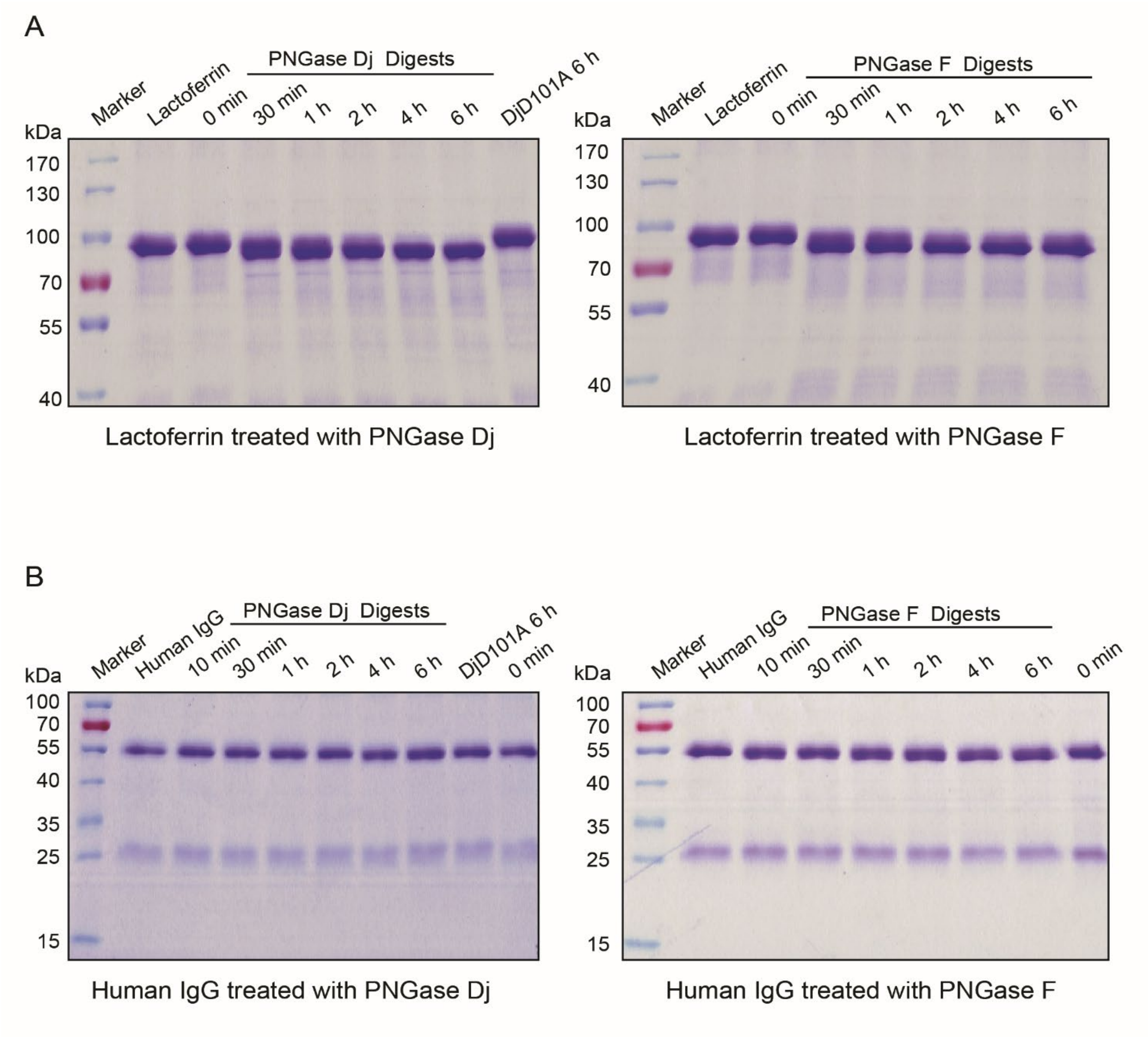

Supplementary Figure 7. Gel-based deglycosylation assays of Lactoferrin **(A)** and human IgG **(B)** using PNGase Dj (left panels) and PNGase F (right panels).

A

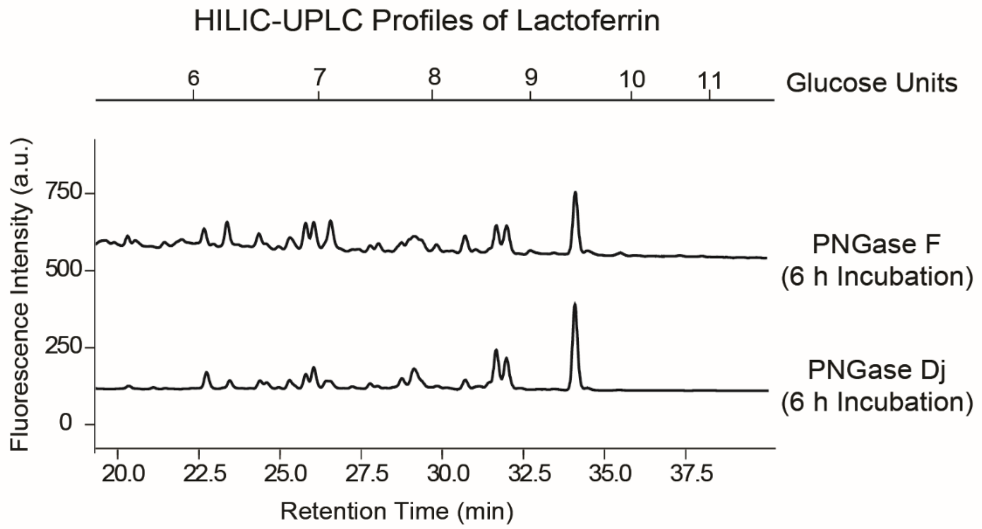

B

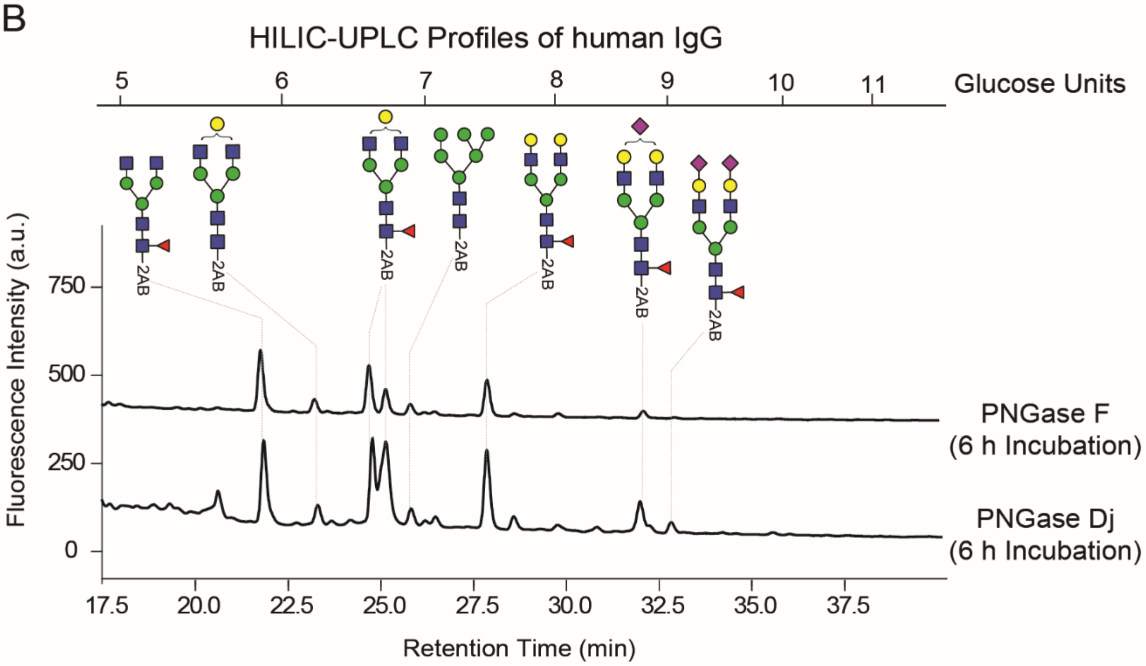

Supplementary Figure 8. HILIC-UPLC analysis of N-glycans released from Lactoferrin **(A)** and human IgG **(B)** using PNGase F and PNGase Dj.

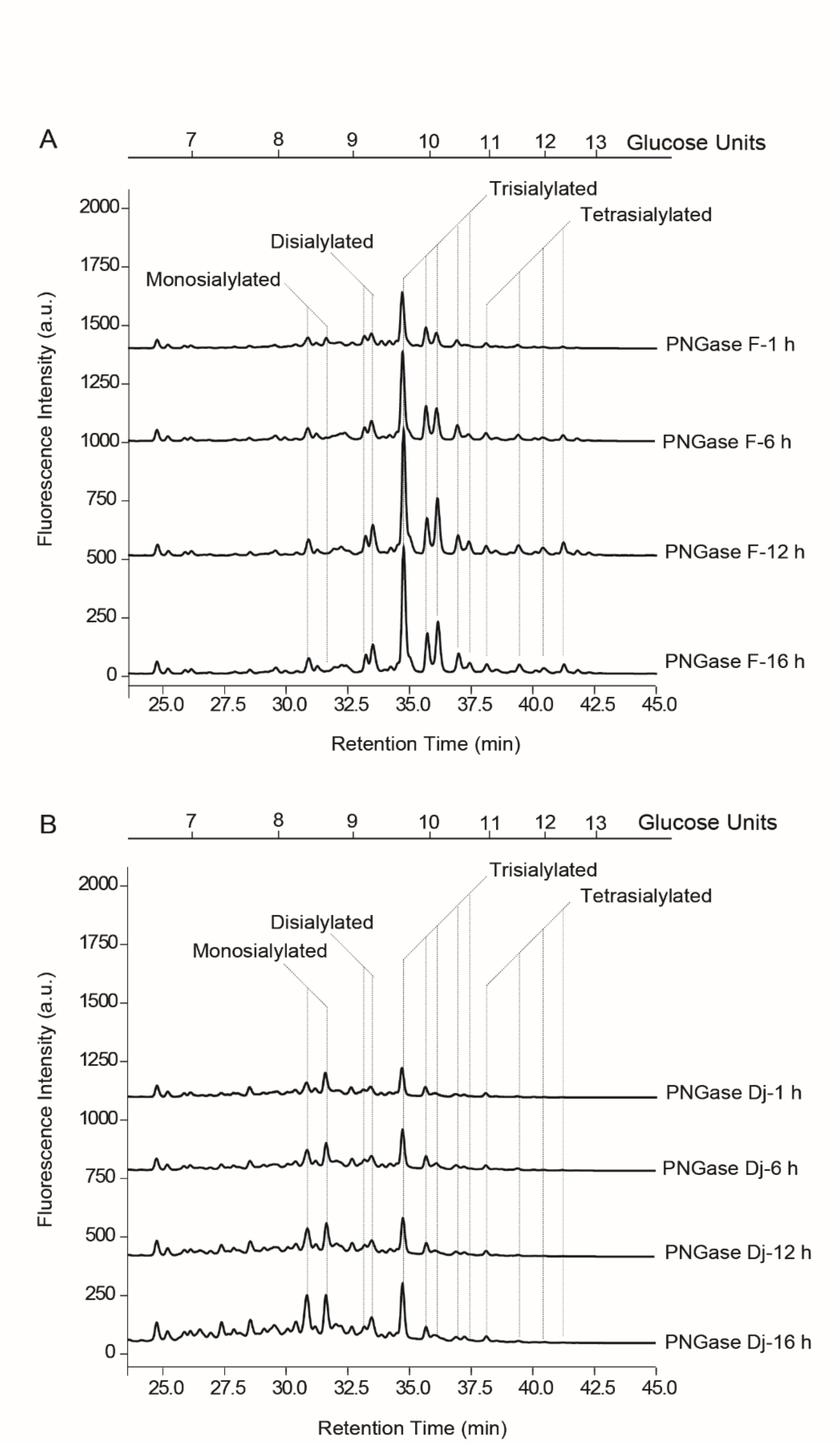

Supplementary Figure 9. HILIC-UPLC analysis of N-glycans released from mouse serum using PNGase F **(A)** and PNGase Dj **(B)**.

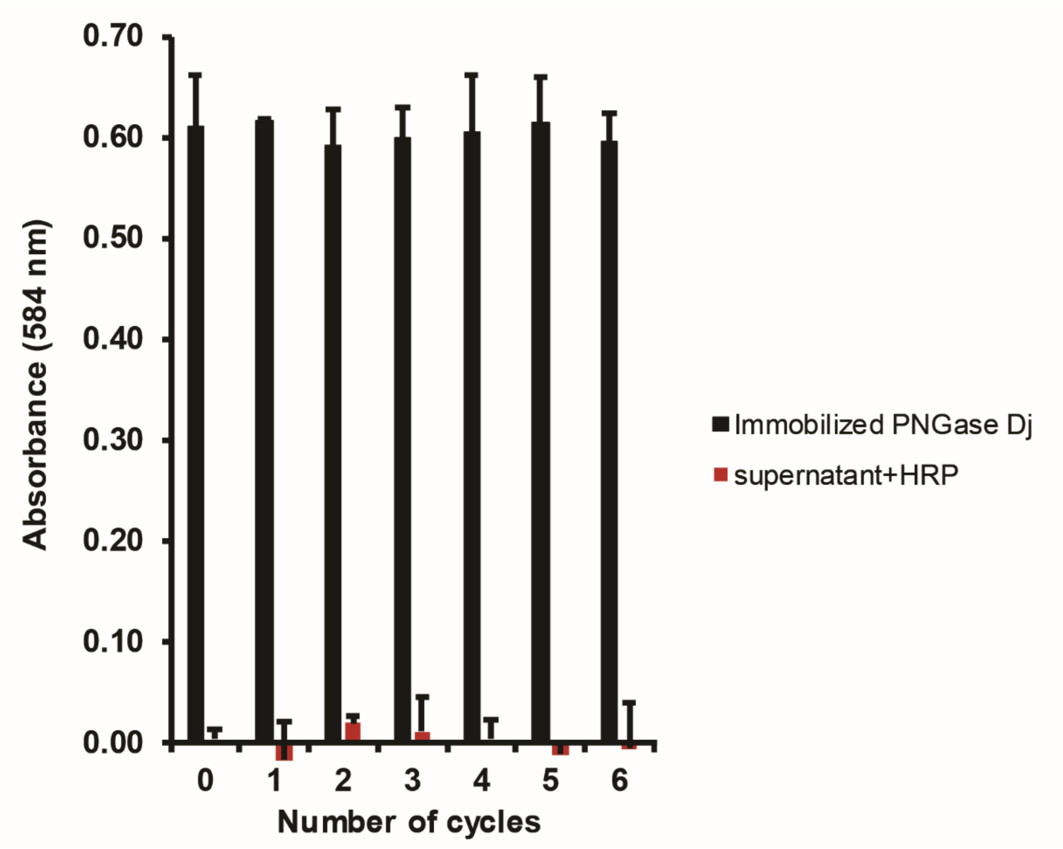

Supplementary Figure 10. Activity of Ni-NTA immobilized PNGase Dj and PNGase activities in reaction buffer supernatants over the course of 6 recycling steps. For measuring the PNGase Dj activity in the supernatant, the Ni-NTA immobilized PNGase Dj in reaction buffer was incubated in a first step without the HRP as a substrate, which was then added to the supernatant after removing the solid Ni-NTA carrier. The data were obtained using the platereader-based photometric PNGase assay and are represented as the mean values and the error bars showing the standard deviation of three independent activity assays.
